# Sensitive peripheral nerve repair during COVID-19 emergency: does the outpatient surgical setting work as well as the operating theater?

**DOI:** 10.1007/s00238-023-02085-x

**Published:** 2023-06-09

**Authors:** Leonardo Garutti, Federico Tamborini, Alessandro Fagetti, Tommaso Baroni, Elisa Bascialla, Andrea Minini, Mario Cherubino, Luigi Valdatta

**Affiliations:** 1 Division of Plastic and Reconstructive Surgery, Department of Biotechnology and Life Sciences, University of Insubria, Via Ravasi 2, 21100 Varese, Italy; 2 Division of Plastic Surgery, Ospedale Di Circolo Fondazione Macchi Varese, Varese, Italy; 3 Microsurgery and Hand Surgery Unit, ASST Settelaghi, Ospedale Di Circolo Fondazione Macchi Varese, Varese, Italy

**Keywords:** Nerve repair, WALANT, Field sterility, COVID-19, Ambulatory hand surgery

## Abstract

**Background:**

Nerve injuries are a common occurrence among hand injuries, which at the time of the COVID-19 emergency, did not appear to have reduced their incidence. The treatment of these injuries is urgent, but the pandemic has led to a reduction in the availability of resources and a consequent reorganization of activities. Principles about Wide-Awake Local Anesthesia No Tourniquet (WALANT) in hand surgery expressed by LaLonde helped hand surgeons to adapt to this new condition by demonstrating a possible outpatient pathway for the treatment of hand traumatic conditions. In the present study, we bring our experience in nerve repair at time of COVID-19 emergency.

**Methods:**

We retrospectively enrolled in this study all patients surgically treated for a peripheral nerve injury (PNI) during the COVID-19 emergency period from March 2020 to March 2022. Demographical, anamnestic, surgical, and postoperative data were recorded and analyzed. Persisting Tinel was set as the primary outcome, while hypoesthesia and other complications as secondary outcomes.

**Results:**

Thirty-six patients have been enrolled. Despite some difference in group homogeneity in term of hypertension and multi-digital involvement, we registered no difference in term of outcomes (*P* > 0.05) between patient operated in surgical theater and in outpatient clinic and between the various techniques of nerve repair employed (*P* > 0.05).

**Conclusions:**

Nerve repair on an outpatient facility is technically feasible and was found in this study to be safe and effective. Compared to hospitalization, the outpatient setting has a more “agile” organization and lower costs, making it preferable in selected cases.

Level of evidence: Level IV, Therapeutic.

## Introduction

COVID-19 emergency has been a worldwide threatening condition who changed our life and habit. The impact of COVID-19 emergency in society and hospital organization is well known, consisting in the “re-”introduction of the concept of quarantine and the reorganization of activities with the suspension of all deferrable procedures [1]. At the peak of the pandemic, this even meant the suspension of all procedures related to the treatment of non-life-threatening conditions. In Italy, this phenomenon occurred especially in the most affected areas such as northern regions (e.g., Lombardy) [2, 3]. In this period, hand injuries and therefore conditions that require urgent treatment, such as peripheral nerve injuries (PNI) [4], did not seem to have reduced their incidence [5]. While the redirection of resources and activities towards the treatment of patients affected by SARS CoV-2 (severe acute respiratory syndrome) has led to a reduction in both economic and human resources dedicated to hand surgery, the need for the treatment of urgent injuries has remained. In Italy, this has led hand surgery units to reorganize their activities [2]. The principles associated with Wide-Awake Local Anesthesia No Tourniquet (WALANT) in hand surgery popularized by Don LaLonde [6] helped hand surgeons to cope with this new situation, opening the way to the outpatient pathway for the treatment of minor traumatic hand conditions. In the present study, we bring our experience in nerve repair at time of COVID-19 emergency and the consequent reorganization of the care pathway, with the aim to evaluate the safety and effectiveness of the outpatient treatment pathway compared to ordinary hospitalization and day surgery. The secondary purpose was to assess which nerve repair technique was the most beneficial.

## Materials and methods

A retrospective review of all patients undergoing surgery during the emergency period of the COVID-19 pandemic (March 2020–March 2022) at “ASST-Settlaghi Circolo e Fondazione Macchi” hospital was performed. Patients with hand PNI who received surgical treatment were selected. The inclusion criteria were as follows: trauma to the hand involving a sensitive peripheral nerve such as common digital nerves, laterodigital nerves, and the superficial branch of the radial nerve. Patients with main nerve injury, such as median and ulnar nerves in the wrist or forearm, and complex or major hand trauma (including amputations) and patients who were clinically unstable at the time of injury were excluded from the study.

Demographic, anamnestic, and surgical data were recorded, such as the surgical course and neurorrhaphy technique, as well as outcomes and postoperative complications. For the analysis, patients were grouped according to the surgical pathway between those operated in outpatient clinical setting and those operated in a surgical theater.

The outpatient clinical setting included a surgical room in which usually small surgery is performed such as small skin or subcutaneous tumor excision. In this setting, sterile draping is limited to the area involved by the lesion. An 8/0 polyamide monofilament suture wire was used for the nerve repair. The surgery was performed under WALANT by two surgeons (one hand surgery–experienced specialist and a resident) under × 3.5 magnification loupes with external help of a nurse. The nerve was then directly repaired and occasionally wrapped with an external sheath of vein conduit or with fibrin glue.

Patients undergoing surgery in the operating room were treated under axillary plexus anesthesia with a tourniquet. Patient were then admitted in ward for postoperative observation. Patients undergoing the outpatient pathway were instead discharged immediately from the hospital after operation was concluded. The surgical technique, instruments, and optical magnification used were the same in both types of pathway.

Oral antibiotic therapy with amoxicillin and clavulanic acid was administered from the time of injury until 5 days postoperatively in all patients, in line with the hospital protocol for penetrating trauma injuries. Also, patients were divided into three groups according to the surgical technique used for nerve repair: direct nerve suture (DNS), direct nerve suture reinforced by a sleeve of fibrin glue (FGS), direct nerve suture with vein wrapping (VW).

Presence of the Tinel sign at 3–6 months at the site of the trauma, indicating a development of neuroma, was set as the primary outcome. Residual hypoesthesia and postoperative complications such as infection, wound dehiscence, and stiffness were considered as secondary outcomes. Hypoesthesia was defined by a static 2-point discrimination test on the fingertips above 8 mm.

Statistical analysis was performed with SPSS® Statistics version 23.0 (IBM SPSS Statistics for Windows; IBM Corp., Armonk, NY, USA) and parametric and non-parametric test such as the chi-square, Fisher, and Mann–Whitney tests with a confidence interval set at 95% (significative *P* value < 0.05). The study was conducted in accordance with the Declaration of Helsinki, with patients’ consensus obtained during the follow-up.

## Results

During the considered period, a total of 36 patients have been retrospectively identified and enrolled in the present study. The demographic, anamnestic, and operative characteristics of the population are described in Table 1.

**Table 1:** Population characteristics

Total patients	36
Age at trauma, mean years (SD)	47.5 (16.76)
Gender (female, male)	7, 29
Smoking	8
Hypertension	7
Diabetes	3
**Damaged nerve**	
Single laterodigital nerve	19
Both laterodigital nerve	5
Common digital nerve	4
Superficial radial branch	8
Associated lesions, *n*	28
Δ-tTR, mean (SD)	3.11 (3.76)
Follow-up, mean days (SD)	135.42 (124.84)

*Δ-tTR* time between trauma and repair

According to surgical pathway, the patients were divided into two groups; among them, 18 underwent surgery in an operative theater (regular hospitalization or day surgery), and the others underwent surgery on an outpatient facility. In Table 2 are compared the characteristics and the outcomes of the two groups. Both groups appear to be uniform, except for hypertension (6 vs 1, *P* = 0.035) and multi-digital trauma (more than one finger involved) (9 vs 2, *P* = 0.011), which were statistically different. No statistically significant difference was found between the two groups in terms of outcome (*P* > 0.05). Among the general complications (8 patients), there were no cases of infection; stiffness was the only complication recorded, with the exception of one case treated on an outpatient basis that developed a retractile scar.

**Table 2:** Surgical pathway

	Operating theater* (18 patients)	Outpatient clinic (18 patients)	*P* value
Age, mean (SD)	40.39 (19.251)	46.56 (13.686)	0.276
Gender			
Female, male	3, 15	4, 14	0.674
Smoking	6	2	0.109
Hypertension	6	1	0.035
Diabetes	2	1	0.546
ASA score ≥ 3	2	0	0.486
Multi-digital trauma	9	2	0.011
Involved nerve			
LD, LDRU, CD, SRB	8, 2, 5, 3	11, 2, 0, 5	0.113
Associated lesions	15	13	0.100
Tendon injury	16	12	0.109
Δ-tTR, mean days (SD)	3.39 (3.68)	2.83 (3.93)	0.664
Outcomes			
Tinel +	6	6	1.00
Hypoesthesia	9	10	0.738
Complications	6	2	0.109

*ASA* American Society of Anesthesiologists, *LD* laterodigital nerve (radial or ulnar), *LDRU* laterodigital nerve radial and ulnar, *CD* common digital nerve, *SRB* superficial radial branch, *Δ-tTR* time between trauma and repair

^*^Day surgery + ordinary admission

Moreover, to evaluate the difference between the surgical technique used for nerve repair, patients were sorted in three groups: DNS (18), FGS (7), VW (11). The groups were similar except for the timing of trauma repair that was delayed in patients in which a vein wrapping was performed (1.72 vs 2.29 vs 5.91, *P* = 0.008). Once again, no difference was found between the outcomes considered (*P* > 0.05) (Table 3).

**Table 3:** Neurrorraphy technique

	DNS (18 patients)	FGS (7 patients)	VW (11 patients)	*P* value
Age, mean (SD)	40.89 (17.92)	43.86 (14.10)	47.45 (16.96)	0.604
Gender				
Female, male	5, 13	2, 5	0, 11	0.148
Smoking	4	2	2	0.875
Hypertension	2	2	3	0.449
Diabetes	2	0	1	0.662
Multi-digital trauma	8	1	2	0.192
Involved nerve				
LD, LDRU, CD, SRB	10, 2, 5, 1	4, 1, 0, 2	5, 1, 0, 5	0.112
Associated lesions	16	5	7	0.453
Tendon injury	2	2	4	0.256
Δ-tTR, mean days (SD)	1.72 (1.638)	2.29 (2.498)	5.91 (5.356)	0.008
Outcomes				
Tinel +	5	2	5	0.592
Hypoesthesia	8	6	5	0.151
Complications	5	3	0	0.075

*DNS* direct nerve suture, *FGS* fibrin glue sleeve, *VW* vein wrapping, *LD* laterodigital nerve (radial or ulnar), *LDRU* laterodigital nerve radial and ulnar, *CD* common digital nerve, *SRB* superficial radial branch, *Δ-tTR* time between trauma and repair

In our study, the time between trauma and repair (Δ-tTR) was found not to be associated to higher complications (*P* = 0.125), hypoesthesia (*P* = 0.655), and development of Tinel (*P* = 0.196).

## Discussion

COVID-19 emergency has been a worldwide catastrophe in terms of economy and lives. In Italy, in order to face the overwhelming amount of patients with COVID-19 infection, hospitals underwent to a structural reorganization. This included a conversion of all the surgical wards to internal medicine wards, a switch of all the activities of healthcare providers (medical, doctors, and nurses) to the treatment of SARS Cov-2 patients with reduction of resources for directing the treatment of other pathologies [3]. From the surgeon’s point of view, the consequence of this reorganization was a drastic reduction in the availability of operating theaters. On the other side, hand injuries did not decrease, nor did the number of patients with traumas requiring urgent surgical treatment [5] such as those with nerve injury.

As promoted by LaLonde and Phillips [6], the surgical treatment of small trauma of the hand can be safely accomplished in a “minor procedure room” without the necessity of a recovery room. This is feasible thanks to the WALANT anesthetic technique, which implies the use of a sort of tumescent technique around and in the site of the operative area with diluted adrenaline and anesthetic solution. The double benefits intrinsic of WALANT are the possibility of covering a large area (i.e., all the hand) and the effective control of bleeding [7].

In our center, the transition to outpatient management of certain types of hand injuries arose from the need to treat patients with non-deferrable conditions at a time when there was no availability of surgical rooms due to the COVID-19 pandemic emergency. In this context, all peripheral hand injuries that did not require a specific instrument (e.g., an optical microscope) were referred to outpatient management. For patients’ safety, the decision to submit them to the outpatient route was subject to their surgical risk, assessed on the basis of comorbidity (ASA score higher than 3), and COVID-19 positivity of the nasopharyngeal swab. In those cases, patients were referred directly to ordinary hospitalization, which only happened in one case.

The primary objective of our study was to evaluate the safety and efficacy of outpatient setting treatment in PNI of the hand. By comparing the two groups of patients (those operated in outpatient context vs those hospitalized) we found that there was no difference in terms of developing complication such as neuroma or infection, with the same efficacy of treatment. Our results regarding infection rate seems to be in line with those reported in literature [8, 9].

Although no cost-analysis was carried out in this study, another consideration is about the cost economic impact of the two pathways. For the same treatment efficacy and safety, outpatient management avoids and significantly reduces the costs associated with patients’ admission to the hospital, the amount of staff involved, and the occupation time of the operating theater. Therefore, for selected patients, we believe that for minor hand traumas, the outpatient setting should be preferable.

Secondly, the surgical technique used for nerve repair was analyzed. No significant difference in outcomes was found between epineural nerve suture, venous bandage, and epineural suture with fibrin glue sheath. The use of fibrin glue to reinforce nerve sutures may be not worth cost-effective. Vein wrapping serves the purpose of isolating and reinforcing the neurorrhaphy, but implies the harvest of a donor vein and increases the operative time. Although the groups in our cases differed in terms of delay in trauma treatment, we found no benefit in using venous or “glue” sleeves. This evidence, in accordance with the literature [10], suggests that classical neurorrhaphy seems to be the simplest and most convenient technique in cases of nerve injuries without significant gaps. Our results may have been influenced by a different timing in the treatment, thereby disadvantaging the VW group that was treated with a higher delay.

In the present study, oral antibiotic therapy has been administered to all patients for two reasons. Although our hospital is a trauma center, minor injuries of the hand are not always immediately evaluated by the hand surgeon, and the surgical assessment and thus accurate wound irrigation may be delayed. Secondly, in our area, these are often work-related injuries, occurring in manual workers in dirty conditions with tools such as saws, or alternatively from animal bites. While literature suggest that antibiotic therapy is not required in small hand trauma [11], this is not for this kind of wounds. For these reasons, in order to prevent the development of infections, in our institute, we prefer to administer a course of antibiotic therapy to all patients with penetrating hand trauma regardless of the surgical pathway.

The limitations of this study are as follows: the retrospective design, the low number of patients enrolled, and the difference in homogeneity found between the groups. Patients included are relatively few, since we wanted to analyze a very specific pattern of lesion such as PNI of the hand within a peculiar time frame. When comparing the surgical pathways (Table 1), a difference in both groups regarding the hypertension prevalence and multi-digital trauma (*P* < 0.05) was found. Hypertension was more prevalent in the hospitalized group. This could be a selection bias between the two groups, given that patients were admitted because they were more “fragile.” However, other comorbidities such as diabetes and the ASA score were comparable in both groups, refuting this hypothesis. Another objection could be the possible influence of hypertension in outcomes. Hypertension can contribute to neuropathy in diabetic patients [12, 13]; however, in literature, the association between hypertension and impairment of nerve recover after repair is not described. In our study, only one patient had a history of a combination of diabetes and hypertension, but did not develop any complications, Tinel or hypoesthesia. In this paper, multi-digital trauma was a selection bias, yet the outcomes were comparable. Involving more than one finger may increase the operating time, and this must be weighted with patient compliance when deciding whether an outpatient procedure is feasible. Further studies are needed to prevent the effect of biases encountered in this study regarding the homogeneity of the population, and to gain a better evaluation on the safety and efficacy of the outpatient surgical pathway.

## Conclusions

Nerve repair on an outpatient setting is technically feasible and was found in this study to be safe and effective, with good functional results and complication incidence. This can be achieved by reproducing a sterile surgical field and taking care to maintain antisepsis during the procedure. Compared to hospitalization, the outpatient setting has a more “agile” organization and lower costs, making it preferable in selected cases. Patient safety is paramount and the selection must be weighed against operational risk and patient compliance.

## Data Availability

The data used for this study are collected in a non-publicly accessible, password-protected excell database.
